# Relation between Mild to Moderate Chronic Kidney Disease and Coronary Artery Disease Determined with Coronary CT Angiography

**DOI:** 10.1371/journal.pone.0047267

**Published:** 2012-10-10

**Authors:** Ivo A. Joosen, Frank Schiphof, Mathijs O. Versteylen, Eduard M. Laufer, Mark H. Winkens, Patricia J. Nelemans, Jeroen P. Kooman, Leonard Hofstra, Joachim E. Wildberger, Tim Leiner

**Affiliations:** 1 Department of Cardiology, Cardiovascular Research Institute Maastricht, Maastricht University Medical Center, Maastricht, The Netherlands; 2 Department of Radiology, Cardiovascular Research Institute Maastricht, Maastricht University Medical Center, Maastricht, The Netherlands; 3 Department of Epidemiology, Maastricht University Medical Center, Maastricht, The Netherlands; 4 Department of Internal Medicine, Maastricht University Medical Center, Maastricht, The Netherlands; 5 Cardiology Center Netherlands, Utrecht, The Netherlands; 6 Department of Radiology, University Medical Center Utrecht, Utrecht, The Netherlands; Ochsner Health System, United States of America

## Abstract

**Background:**

Both end-stage and milder stages of chronic kidney disease (CKD) are associated with an increased risk of adverse cardiovascular events. Several studies found an association between decreasing renal function and increasing coronary artery calcification, but it remains unclear if this association is independent from traditional cardiovascular risk factors. Therefore, the aim of this study was to investigate whether mild to moderate CKD is independently associated with coronary plaque burden beyond traditional cardiovascular risk factors.

**Methods:**

A total of 2,038 patients with symptoms of chest discomfort suspected for coronary artery disease underwent coronary CT-angiography. We assessed traditional risk factors, coronary calcium score and coronary plaque characteristics (morphology and degree of luminal stenosis). Patients were subdivided in three groups, based on their estimated glomerular filtration rate (eGFR) Normal renal function (eGFR ≥90 mL/min/1.73 m^2^); mild CKD (eGFR 60–89 mL/min/1.73 m^2^); and moderate CKD (eGFR 30–59 mL/min/1.73 m^2^).

**Results:**

Coronary calcium score increased significantly with decreasing renal function (P<0.001). Coronary plaque prevalence was higher in patients with mild CKD (OR 1.83, 95%CI 1.52–2.21) and moderate CKD (OR 2.46, 95%CI 1.69–3.59), compared to patients with normal renal function (both P<0.001). Coronary plaques with >70% luminal stenosis were found significantly more often in patients with mild CKD (OR 1.67 (95%CI 1.16–2.40) and moderate CKD (OR2.36, 95%CI 1.35–4.13), compared to patients with normal renal function (both P<0.01). After adjustment for traditional cardiovascular risk factors, the association between renal function and the presence of any coronary plaque as well as the association between renal function and the presence of coronary plaques with >70% luminal stenosis becomes weaker and were no longer statistically significant.

**Conclusion:**

Although decreasing renal function is associated with increasing extent and severity of coronary artery disease, mild to moderately CKD is not independently associated with coronary plaque burden after adjustment for traditional cardiovascular risk factors.

## Introduction

Coronary artery disease (CAD) remains one of the leading causes of morbidity and mortality in developed countries. In 2007, a total of 406,351 people died due to CAD in the United States. Each year, an estimated 785,000 Americans will suffer a new coronary attack, and 470,000 will have a recurrent attack. It is estimated that an additional 195,000 silent first myocardial infarctions occur each year [1]. Chronic kidney disease (CKD) is also recognized as a major worldwide public health problem, as evidenced by an increasing incidence and prevalence of patients with kidney failure requiring renal replacement therapy, with poor outcomes and high costs [2]. Nearly 26 million people (13%) in the United States have CKD, and most are undiagnosed, while another 20 million Americans are at increased risk for CKD [3], [4].

Several studies have found an increased prevalence of CAD, congestive heart failure and left ventricular hypertrophy in patients with end-stage CKD [5], [6]. More recently, earlier stages of CKD have also been associated with a worse prognosis in patients with as well as without known CAD [7], [8]. In order to further elucidate the interplay between renal function and symptomatic CAD, more detailed knowledge about coronary artery disease burden is desired in patients with different degrees of renal impairment.

Coronary computed tomographic angiography (CCTA) is a noninvasive diagnostic imaging tool, which provides precise information regarding the presence and extent of coronary calcium deposits as well as coronary plaque localization, degree of luminal stenosis and plaque morphology. Several studies already found an inverse association between renal function and coronary artery calcification, but it remains unclear if this association is independent from traditional cardiovascular risk factors [9]–[14].

Therefore, the aim of this retrospective cross-sectional study was to investigate whether mild to moderate chronic kidney disease is independently associated with coronary plaque burden beyond traditional cardiovascular risk factors.

## Methods

### Ethics Statement

This study was approved by the Institutional Review Board (IRB) and Ethics Committee at the Maastricht University. Involved data were collected on a routine basis within the Maastricht Biomarker CT study. Analyses were carried out retrospectively. Informed consent was not obtained from individual patients because the data were analyzed anonymously in accordance with IRB guidelines. The study complies with the ethical principles of the Helsinki Declaration of 1964, revised by the World Medical Organization in Edinburgh in 2000.

### Study Population

**Figure 1:**
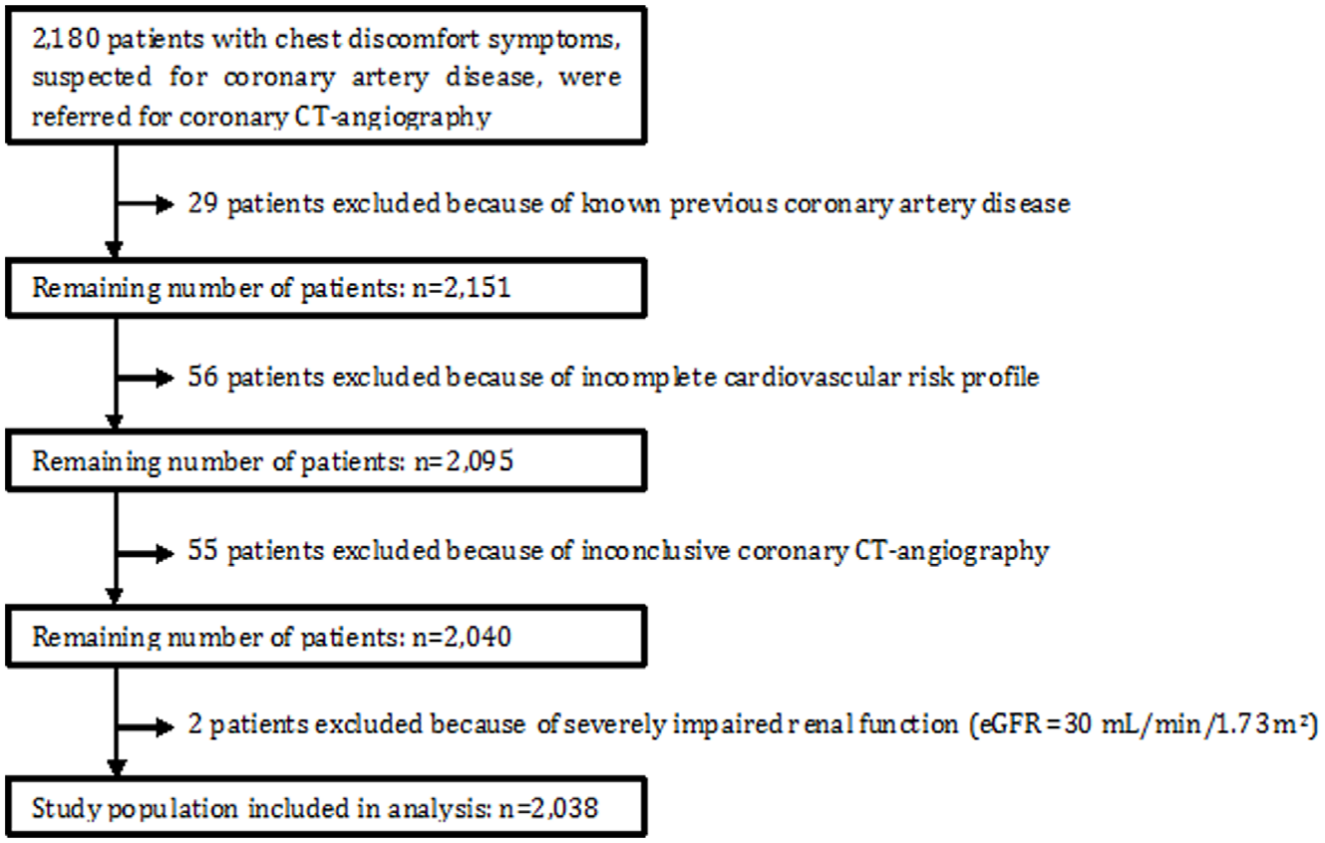
Flowchart of the study design.

We studied 2,180 consecutive adult patients who were referred from the cardiology outpatient department for CCTA because of chest discomfort symptoms, suspected for CAD. All scans were performed in the Maastricht University Medical Center between 2008 and 2012 as part of the diagnostic work-up. Included were patients with a recently (not older than 1 month) measured creatinine, who underwent coronary calcium scoring (CCS) scan as well as CCTA. Excluded were patients with a known history of CAD, patients with missing data regarding their cardiac risk profile, patients with an inconclusive scan and patients with severely impaired renal function, defined as an estimated glomerular filtration rate (eGFR) ≤30 mL/min/1.73 m^2^ (Figure 1).

### Traditional Cardiovascular Risk Factors

Traditional cardiovascular risk factors were collected prior to the scan. Patients were classified as smoker if they were current smoker. A positive family history was defined as having a first-degree relative with a history of myocardial infarction or sudden cardiac death before the age of sixty. Patients were classified as diabetic if diabetes mellitus was diagnosed by a medical doctor according to the guidelines [15]. The body mass index (BMI) is defined as the individual’s body mass (kg) divided by the square of the height (m^2^).

### Chronic Kidney Disease (CKD) Categories

For all patients, the eGFR was calculated using the CKD-EPI Equation: eGFR = 141×min(Scr/κ,1)^α^×max(Scr/κ,1)^−1.209^×0.993^Age^×1.018 [if female]×1.159 [if black], where Scr is serum creatinine, κ is 0.7 for females and 0.9 for males, α is -0.329 for females and -0.411 for males, min indicates the minimum of Scr/κ or 1, and max indicates the maximum of Scr/κ or 1 [16]. The eGFR is expressed as mL/min/1.73 m^2^. Patients were divided into three categories: normal renal function (eGFR ≥90 mL/min/1.73 m^2^), mildly impaired renal function (eGFR 60–89 mL/min/1.73 m^2^) and moderately impaired renal function (eGFR 30–59 mL/min/1.73 m^2^) based on the National Kidney Foundation Kidney Disease Outcomes Quality Initiative practice guidelines for classification [17].

### Coronary CT-angiography Protocol

Scans were performed using a 64–slice multidetector-row CT-scanner (Brilliance 64; Philips Healthcare, Best, The Netherlands, n = 1,026) or a dual-source CT-scanner (Somatom Definition Flash, Siemens Medical Solutions, Forchheim, Germany, n = 1,012). Data acquisition parameters for the Brilliance 64 were a 64×0.625 mm slice collimation, a gantry rotation time of 420ms and a tube voltage of 80–120 kV, depending on the patient’s height and weight. Data acquisition parameters for the Somatom Definition Flash were a 2×128×0.600 mm slice collimation, a gantry rotation time of 280 ms and a tube voltage of 80–120 kV. Patient preparation was identical for both CT-scanners. Patients received 50 mg Metoprolol tartrate (AstraZeneca, Zoetermeer, The Netherlands) orally, two hours before CCTA. When indicated, an additional dose of 5–20 mg Metoprolol tartrate was administered intravenously to lower the heart rate to <60 beats per minute (bpm). 0.8 mg Nitroglycerin spray (Pohl-Boskamp, Hohenlockstedt, Germany) was given sublingually just prior to CCTA. Heart rate and ECG were monitored during CCTA.

A non-enhanced scan was performed to determine the CCS using the Agatston method [18]. Subsequently, CCTA was performed using 75–120 mL of contrast agent (Xenetix 350; Guerbet, Roissy CdG Cedex, France or Ultravist 300; Bayer Pharma AG, Berlin, Germany), which was injected in the antecubital vein at a rate of 5.2–7.4 mL/s, directly followed by 40 mL intravenous saline (6.0 mL/s) using a dual-head power injector (Medrad Inc, Indianola, Pennsylvania, USA). Both the amount of contrast agent as well as the flow rate were dependent on individual patient characteristics.

Scan protocols were different for both CT-scanners. For the Brilliance 64, a prospectively gated “Step and shoot” protocol was used in all patients with a stable heart rate <65 bpm. In patients with a heart rate >65 bpm, we used a retrospectively gated “Helical” protocol with dose modulation to obtain the best image quality at minimal radiation dose [19], [20]. For the Somatom Definition Flash, a prospectively gated high pitch spiral “Flash” protocol was used in patients with a stable heart rate <60 bpm. In patients with a stable heart rate between 60–90 bpm, we used a prospectively gated axial “Adaptive sequence” protocol. In patients with a heart rate >90 bpm or in case of an irregular heart rhythm, we used a retrospectively gated “Helical” protocol with dose modulation.

### Coronary CT-angiography Analysis

Dedicated workstations (Philips Brilliance Workspace Portal and Siemens Syngo MultiModality Workplace) were used to assess the source images. Scans were independently analyzed by a cardiologist and a radiologist, both with level III expertise in coronary CT-angiography and both blinded for patient details. In case of disagreement, consensus was reached by reviewing findings jointly.

CCS was expressed as the Agatston score using dedicated calcium scoring software with a threshold of 130 Hounsfield units (HU). The coronary artery tree was analyzed for the presence and severity of CAD, according to the classification of the American Heart Association [21]. Coronary plaques were defined as visible structures within or adjacent to the coronary artery lumen, which could be clearly distinguished from the vessel lumen and the surrounding pericardial tissue. Plaques were categorized as calcified (exclusively content >130 HU), non-calcified (exclusively content <130 HU) or mixed (characteristics of both calcified and non-calcified plaques). The degree of CAD was visually estimated and classified as absent (no luminal stenosis), mild (<50% luminal stenosis), moderate (50–70% luminal stenosis) or severe (>70% luminal stenosis) [22].

### Statistical Analyses

Categorical baseline characteristics are expressed as percentages, while continuous variables are expressed as means (standard deviation; SD) or as median (interquartile range; IQR). To test differences between the CKD groups for statistical significance, we used analysis of variance (ANOVA).

Odds ratios (OR) with corresponding 95% confidence intervals (CI) were used to quantify the association between renal function and characteristics of coronary plaques, where patients with normal renal function (eGFR ≥90 mL/min/1.73 m^2^) were used as reference category (OR = 1.00). To adjust for traditional risk factors, multivariable logistic regression analysis was performed with presence of any plaque and presence of severe plaque as dependent variables. Included in the models were traditional cardiovascular risk factors as well as eGFR as categorical variable, where patients with normal renal function were used as reference category.

The smallest detectable OR (alpha = 5%, power = 80%) for the detection of any coronary plaque was 1.3 for patients with mild CKD and 1.6 for patients with moderate CKD (proportion exposed among controls = 50%). For the detection of severe coronary plaque, the smallest detectable OR (alpha = 5%, power = 80%) was 1.6 for patients with mild CKD and 2.1 for patients with moderate CKD, respectively (proportion exposed among controls = 6%). A P-value <0.05 was considered significant. Statistical analyses were performed using SPSS software (version 19.0, SPSS Inc., Chicago, IL, USA).

## Results

### Study Population

Patients with chest discomfort symptoms, suspected for CAD were studied. From 2,180 patients, 29 patients had a history of CAD, 56 patients had missing data concerning their risk profile, 55 patients had an inconclusive CCTA due to poor image quality because of movement and/or breathing artifacts and 2 patients suffered from severely impaired renal function (Figure 1). Baseline characteristics of the remaining 2,038 patients are listed in Table 1. A total of 1,778 patients underwent a prospectively gated scan protocol (mean radiation dose 3.2 mSv), whereas 260 patients underwent a retrospectively gated scan protocol (mean radiation dose 11.1 mSv).

**Table 1:** Baseline characteristics of the study population stratified by eGFR.

		eGFR (mL/min/1.73m^2^)			
Baseline characteristics	All	≥90	60–89	30–59	P value
	(n = 2,038)	(n = 745)	(n = 1,138)	(n = 155)	
Age, mean (SD), years	56 (11)	50 (10)	59 (9)	66 (9)	<0.001
BMI, mean (SD), kg/m^2^	26.8 (4.5)	26.5 (4.7)	26.8 (4.2)	27.7 (4.8)	0.01
Male gender, %	51.5	56.1	51.0	32.9	<0.001
Diabetes mellitus, %	7.5	7.5	6.6	13.5	<0.01
Smoking, %	22.3	28.6	19.1	15.5	<0.001
Positive family history, %	37.0	41.1	36.1	23.9	<0.001
Systolic BP, mean (SD), mmHg	142 (19)	138 (18)	143 (19)	149 (21)	<0.001
Total cholesterol, mean (SD), mg/dL	213 (46)	209 (43)	213 (43)	201 (50)	<0.01
HDL-C, mean (SD), mg/dL	50 (19)	50 (19)	50 (19)	50 (19)	0.71
LDL-C, mean (SD), mg/dL	131 (39)	131 (39)	135 (39)	124 (46)	<0.01
Triglycerides, mean (SD), mg/dL*	159 (106)	159 (106)	159 (106)	151 (80)	0.92
Glucose, mean (SD), mg/dL**	105 (34)	106 (27)	105 (27)	108 (36)	0.50
Calcium score, median (IQR)	4 (0–95)	0 (0–40)	10 (0–115)	38 (0–220)	<0.001
Presence of any plaque, %	59.6	49.8	64.5	71.0	<0.001
Presence of moderate plaque, %	22.7	19.1	24.2	29.0	<0.01
Presence of severe plaque, %	8.4	5.9	9.5	12.9	<0.01

Baseline characteristics, stratified by eGFR. eGFR, estimated glomerular filtration rate; BMI, body mass index; BP, blood pressure; HDL-C, high-density lipoprotein cholesterol; LDL-C, low-density lipoprotein cholesterol. * Triglycerides measured in n = 1,786. ** Glucose measured in n = 1,568.

Among the 2,038 patients, 745/2,038 (36.6%) had normal renal function, 1,138/2,038 (55.8%) had mildly impaired renal function, and 155/2,038 (7.6%) had moderately impaired renal function. Compared to patients with normal renal function, patients with mild or moderately impaired renal function were older, had a higher mean body mass index and a higher mean systolic blood pressure. On the other hand, they were less likely to have a positive family history and they were less often male and smoker, Table 1.

### Coronary Calcium Score and Coronary CT-angiography

**Figure 2:**
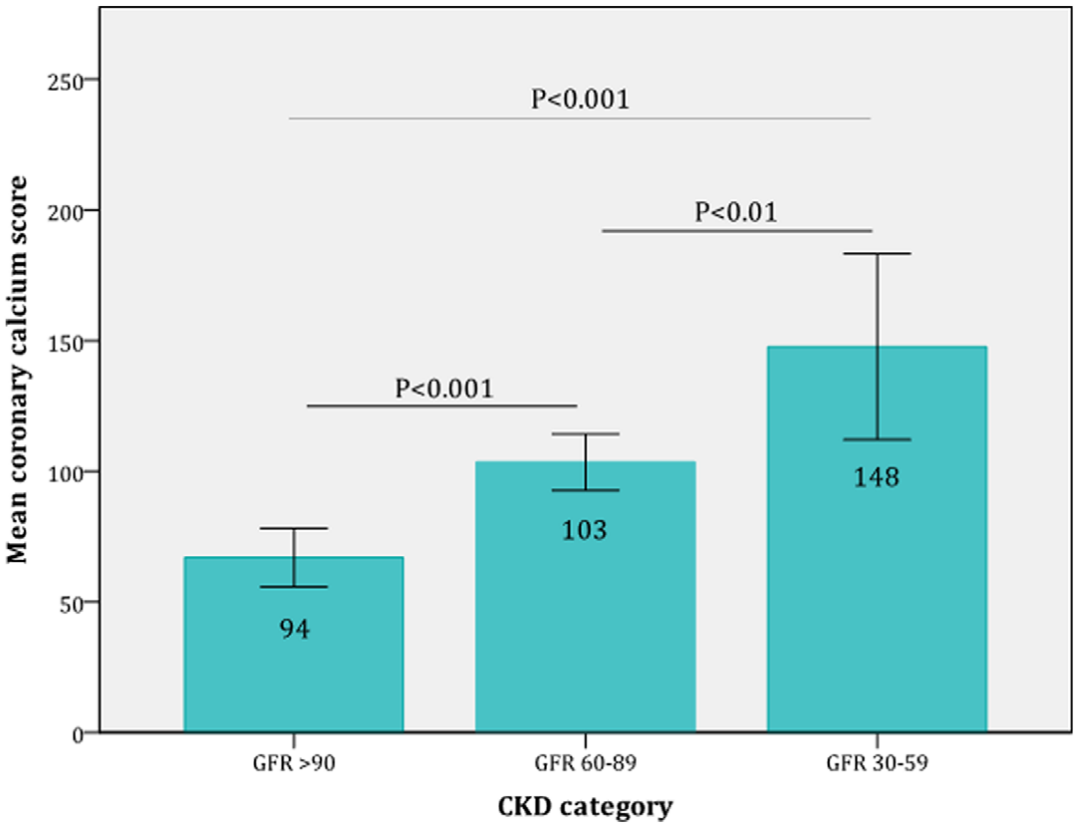
Mean coronary calcium score in the different CKD-categories. The graph clearly shows that the mean calcium score increased significantly with decreasing renal function.

The mean CCS increased significantly with decreasing renal function. The mean CCS was 94 in patients with normal renal function, 103in patients with mildly impaired renal function and 148 in patients with moderately impaired renal function (all P<0.01), (Figure 2).

Coronary plaques were found in 1,215 of 2,038 patients (59.6%). Patients with mildly (OR 1.83, 95%CI 1.52–2.21) or moderately (OR 2.46, 95%CI 1.69–3.59) impaired renal function significantly more often harbored coronary plaques compared to patients with normal renal function (both P<0.001). This observation extended to the presence of severe coronary plaques (luminal stenosis >70%), which were also found significantly more often in patients with mildly (OR 1.67, 95%CI 1.16–2.40) or moderately (OR 2.36, 95% CI 1.35–4.13) impaired renal function compared to patients with normal renal function (both P<0.01), Table 2.

**Table 2:** Presence of any, moderate and severe coronary plaque in patients, stratified by eGFR.

(n = 2,038)		eGFR ≥90	eGFR 60–89	eGFR 30–59
Any plaque	OR (95%CI)	1.00 (reference)	1.83 (1.52–2.21)	2.46 (1.69–3.59)
	P value	–	<0.001	<0.001
Severeplaque	OR (95%CI)	1.00 (reference)	1.67 (1.16–2.40)	2.36 (1.35–4.13)
	P value	–	<0.01	<0.01

Odds ratios for the presence of any and severe coronary plaques, stratified by eGFR. Patients with normal renal function (eGFR ≥90 mL/min/1.73 m^2^) were used as reference category (OR = 1.00).

As expected, patients with mild (OR 1.70, 95%CI 1.28–2.26, P<0.001) or moderately (OR 1.68, 95%CI 1.01–2.79, P = 0.05) impaired renal function had significantly higher proportions of calcified plaques, while there were no significant differences regarding the presence of mixed and non-calcified plaques, Table 3.

**Table 3:** Plaque morphology in patients, stratified by eGFR.

(n = 1,215)		eGFR ≥90	eGFR 60–89	eGFR 30–59
Calcified plaque	OR (95%CI)	1.00(reference)	1.70 (1.28–2.26)	1.68 (1.01–2.79)
	P value	–	<0.001	0.05
Mixed plaque	OR (95%CI)	1.00(reference)	1.00 (0.78–1.28)	1.02 (0.66–1.56)
	P value	–	0.98	0.94
Non-calcifiedplaque	OR (95%CI)	1.00(reference)	0.91 (0.70–1.19)	0.90 (0.57–1.43)
	P value	–	0.48	0.66

Odds ratios for plaque morphology, stratified by eGFR. Patients with normal renal function (eGFR ≥90 mL/min/1.73 m^2^) were used as reference category (OR = 1.00).

### Correction for Traditional Cardiovascular Risk Factors

The results of multivariable logistic regression analysis are presented in Table 4. Age, male gender, smoking, diabetes mellitus, systolic blood pressure and positive family history are independent risk factors for the presence of any coronary plaque. Age, male gender and smoking remain the only independent risk factors for the presence of severe coronary plaques.

**Table 4:** Multivariable logistic regression of risk factors for the presence of any and severe coronary plaque.

Characteristic	Presence of any plaque			Presence of severe plaque		
	OR	95%CI	P value	OR	95%CI	P value
Age	1.11	1.09–1.12	<0.001	1.06	1.04–1.08	<0.001
Gender (male = 1)	2.85	2.30–3.52	<0.001	3.18	2.22–4.57	<0.001
Smoking (yes = 1)	1.94	1.51–2.50	<0.001	1.84	1.27–2.66	0.001
Diabetes mellitus (yes = 1)	1.81	1.18–2.78	<0.01	0.64	0.32–1.29	0.21
Systolic blood pressure	1.01	1.01–1.02	<0.001	1.00	0.99–1.01	0.48
Fam. history (positive = 1)	1.37	1.11–1.69	<0.01	1.38	0.98–1.94	0.07
Total cholesterol	0.98	0.89–1.07	0.58	1.00	0.87–1.15	1.00
eGFR ≥90 mL/min/1.73 m^2^	1.00	(reference)	–	1.00	(reference)	–
eGFR 60–89 mL/min/1.73 m^2^	0.92	0.73–1.15	0.45	1.21	0.81–1.80	0.36
eGFR 30–59 mL/min/1.73 m^2^	0.75	0.48–1.18	0.21	1.52	0.80–2.90	0.21

Multivariable logistic regression analysis was performed with known traditional cardiovascular risk factors as well as the CKD categories. Patients with normal renal function (eGFR ≥90 mL/min/1.73 m^2^) were used as reference category (OR = 1.00).

After adjustment for traditional cardiovascular risk factors, the association between impaired renal function (expressed by eGFR as categorical variable where patients with normal renal function were used as reference category) and the presence of any coronary plaque as well as the association between impaired renal function and the presence of severe coronary plaque becomes weaker and were no longer statistically significant.

## Discussion

Our results demonstrate that patients with mild or moderately impaired renal function have a higher coronary plaque burden including a higher prevalence of severe coronary plaques compared to patients with normal renal function. Moreover, coronary plaques in patients with impaired renal function exhibit an increased degree of calcification. However, after adjustment for traditional cardiovascular risk factors, mild to moderately impaired renal function is not an independent risk factor for the presence of any coronary plaque nor for the presence of severe plaques. This means that variations in coronary plaque burden in this study can be explained by variations in traditional cardiovascular risk factors.

Our study was motivated by the paucity of data regarding the association between mild to moderately impaired renal function and coronary plaque burden. Several studies investigated the association between CKD and coronary artery calcification [13]–[18]. Although all studies found that impaired renal function was associated with an increasing coronary calcium score, it still remains unclear whether this association is independent from traditional cardiovascular risk factors. Some of these studies found an independent association [13]–[15], while other studies did not [16]–[18]. CCS is a marker for atherosclerotic plaque burden and has been shown to be a predictor for the occurrence of myocardial infarction and cardiovascular death [23]. On the other hand, in case of a CCS of zero, it is still possible to have a so called ‘vulnerable’ plaque, which may rupture and cause an acute coronary event [24]. By means of contrast enhanced CT-angiography, it is possible to visualize these non-calcified plaques. Therefore, we were also able to focus on plaque morphology and degree of luminal stenosis, instead of only using CCS as determinant of CAD. However, in our study, non-calcified plaques were not found more often in patients with an impaired renal function compared to patients with a normal renal function.

At present, a limited number of reports have been published regarding the relationship between impaired renal function and coronary plaque morphology. Cho et al. studied 4,297 asymptomatic subjects undergoing CCTA as part of a general health evaluation. They found that subjects with early CKD (eGFR ≥45 mL/min/1.73 m^2^) had significantly higher prevalence of (obstructive) CAD and CCS >100 compared to subjects without CKD. However, after adjustment for proteinuria and other traditional risk factors, there was no significant association between a decrease in eGFR and the risk of (obstructive) CAD or CCS >100 [25]. Although these findings are in line with our results, the main difference is that our population consists of symptomatic patients with suspected CAD, whereas Cho et al. studied asymptomatic subjects.

Also other studies did not found an independent correlation between CKD and coronary plaque burden [26] or did not adjust for traditional cardiovascular risk factors [27].

The search for novel markers that better predict cardiovascular events in patients with impaired renal function is of considerable clinical interest as it may lead to improved strategies to prevent major adverse cardiovascular events (MACE). Recently, Clase et al. examined the contribution of eGFR and urinary albumin-creatinine ratio beyond traditional cardiovascular risk factors in a large cohort of patients with high cardiovascular risk. They conclude that eGFR as well as the urinary albumin-creatinine ratio add only little to traditional cardiovascular risk factors. However, in contrast to our study, their study outcomes were all-cause mortality and long-term dialysis [28]. It would be of interest to investigate the possible additional contribution of renal impairment over traditional cardiovascular risk factors in the prediction of MACE instead of all-cause mortality and long-term dialysis. Because of the relatively short follow-up time, we did not yet focus on the cardiovascular event rate in our population.

Question remains what precise mechanism can explain the relationship between CKD and MACE. It is well known that traditional risk factors, such as age, hyperlipidemia, smoking and hypertension are associated with the development of both CAD and CKD. However, non-traditional risk factors like albuminuria, proteinuria, homocysteinemia and elevated levels of uric acid are also established factors for the progression of renal disease. Other factors which are suggested in the literature to contribute to this mechanism are anemia, oxidative stress, derangements in calcium-phosphate homeostasis, inflammation and conditions promoting coagulation, which are all associated with accelerated atherosclerosis and endothelial dysfunction [29]–[34].

This study is among the first coronary CT-angiography studies in which various degrees of CKD were compared with coronary plaque characteristics in patients with symptoms of chest discomfort, suspected for CAD. In order to calculate the eGFR, we used the CKD-EPI equation instead of other equations like the Cockcroft-Gault equation or the Modification of Diet in Renal Disease (MDRD) equation since the CKD-EPI equation intends to be more generalizable across various clinical settings [16].

Our study has several limitations that merit comment. First, the eGFR was based on a single creatinine measurement. We did not take information into account regarding the course of renal function over time. This may have influenced the results since the serum creatinine concentration depends on various factors such as physical activity, body weight and muscle mass. However, we only used creatinine values less than 1 month old. Second, we did not have information regarding proteinuria and albuminuria because we did not collect urine samples. Third, although CCTA is a well-established imaging technique for detection of coronary plaques, the functional relevance of the plaques remains unsubstantiated. Fourth, this study was performed in outpatient department patients presenting with symptoms of chest discomfort. Results in the general population might have been different. Finally, we were not able yet to investigate patient outcomes because of the relatively short follow-up time.

In conclusion, although decreasing renal function is associated with increased extent and severity of coronary artery disease in patients with symptoms of chest discomfort, mild to moderate CKD is not independently associated with coronary plaque burden after adjustment for traditional cardiovascular risk factors.
